# The role of E3 ubiquitin ligases and deubiquitinases in bladder cancer development and immunotherapy

**DOI:** 10.3389/fimmu.2023.1202633

**Published:** 2023-05-05

**Authors:** Xuemei Wang, Ying Zhang, Yao Wu, Hongjing Cheng, Xueju Wang

**Affiliations:** Department of Pathology, China-Japan Union Hospital, Jilin University, Changchun, Jilin, China

**Keywords:** E3 ligases, bladder cancer, immunotherapy, resistance, ubiquitination

## Abstract

Bladder cancer is one of the common malignant urothelial tumors. Post-translational modification (PTMs), including ubiquitination, acetylation, methylation, and phosphorylation, have been revealed to participate in bladder cancer initiation and progression. Ubiquitination is the common PTM, which is conducted by E1 ubiquitin-activating enzyme, E2 ubiquitin-conjugating enzyme and E3 ubiquitin-protein ligase. E3 ubiquitin ligases play a key role in bladder oncogenesis and progression and drug resistance in bladder cancer. Therefore, in this review, we summarize current knowledge regarding the functions of E3 ubiquitin ligases in bladder cancer development. Moreover, we provide the evidence of E3 ubiquitin ligases in regulation of immunotherapy in bladder cancer. Furthermore, we mention the multiple compounds that target E3 ubiquitin ligases to improve the therapy efficacy of bladder cancer. We hope our review can stimulate researchers and clinicians to investigate whether and how targeting E3 ubiquitin ligases acts a novel strategy for bladder cancer therapy.

## Introduction

Bladder cancer is one of the common malignant tumors worldwide (1). It was estimated that there are 82,290 new cases and 16,710 deaths in 2023 in the United States. In men, bladder cancer was the eight-leading cause of cancer-associated death in the United States (2). In the world, there were approximately 573,278 new cases with bladder cancer and 212,536 deaths due to this disease (3, 4). Tobacco smoking could be a reason for bladder cancer incidence. In addition, some risk factors, such as chemicals and aromatic amines, arsenic contamination and aluminum, could increase the bladder cancer development. The treatments of bladder cancer often include endoscopic resection, chemotherapy, radiation, intravesical immunotherapy and combination therapy (5–7). The gold standard therapy for MIBC was chemotherapy and radical cystectomy. Bladder-sparing trimodal therapy is also available for MIBC patients. Chemoimmunotherapy is the key strategy for bladder cancer with metastatic feature (8). The treatment of immunotherapy and immune checkpoint inhibitors has not shown the good efficacy in bladder cancer patients (9). However, bladder cancer exhibits immune evasion and poor outcomes, suggesting that novel therapies need to be developed for treating bladder cancer (10).

Several genes have been known to regulate development and aggressiveness in bladder cancer, including Wnt, STAT3, PI3K, AKT, mTOR and PTEN (11–15). For instance, monocarboxylate transporter isoform 1 (MCT1) has been found to govern aggressive and metabolic phenotypes in bladder cancer because higher expression of MCT1 was associated with lymph node, poor survival and distant metastasis (16, 17). Silencing of MCT1 blocked proliferation, invasion, migration and altered the expression of EMT-associated proteins (16). MCT1, MCT4 and CD147 displayed a prognostic implication and a potential role in bladder cancer metabolism (18). MCT1 and CD147 also participated in cisplatin resistance and tumor aggressiveness in bladder cancer (19). In addition, some proteins could be post-translationally modified, including phosphorylation, ubiquitination, acetylation, glycosylation, methylation and SUMOylation (20). Post-translational modification (PTM) has been known to govern tumorigenesis and progression in various cancer types, including bladder cancer (21, 22).

PTMs include ubiquitination, acetylation, phosphorylation, methylation, hydroxylation, lipidation, palmitoylation, and glycosylation (23–27). Autophagy-lysosome pathway and the ubiquitin-proteasomal system (UPS) are common PTMs to control protein stability (28, 29). Ubiquitination is an ATP-mediated process: an E1 ubiquitin-activating enzyme activates ubiquitin, E2 ubiquitin-conjugating enzyme links ubiquitin via a transesterification reaction, E3 ubiquitin-protein ligase makes the binding between E2 enzyme and substrate proteins, leading to ubiquitin transfer from E2 to the specific substrate (30, 31). E3 ubiquitin ligases are critically involved in oncogenesis and progression as well as drug resistance in bladder cancer (32, 33). Targeting E3 ubiquitin ligases has demonstrated to be a novel approach for bladder cancer therapy (34). In this review, we summarize current knowledge regarding the roles of E3 ubiquitin ligases in bladder oncogenesis. Furthermore, we discuss the insights of E3 ubiquitin ligases in regulation of immunotherapy in bladder cancer. Moreover, we highlight the efforts on targeting E3 ubiquitin ligases to improve the efficacy of bladder cancer treatments. We hope our review can encourage researchers to explore how can improve the benefit of bladder cancer therapy via targeting E3 ubiquitin ligases.

## Deubiquitinases in bladder cancer

### USP2a

USP2a has been reported to regulate oncogenesis and progression in a variety of human cancers (35–37). USP2a mRNA expression was reduced in bladder cancer tissues compared with age-matched bladder tissues, and USP2a mRNA expression was decreased in higher stage of MIBC (38). Kim et al. found that USP2a increased tumor progression in part via regulation of cyclin A1 in bladder cancer (39). Specifically, overexpression of USP2a increased cell invasion, migration, chemotherapeutic drug resistance and proliferation. Downregulation of USP2a showed the opposite effects in bladder cancer. USP2a overexpression increased the Erk/MAPK phosphorylation after HB-EGF stimulation in T24 cells. Overexpression of USP2a in T24 cells caused more resistance to cisplatin-induced apoptosis due to inhibition of the cleaved form of PARP (c-PARP). USP2a interacted with cyclin A1 and blocked the ubiquitination of cyclin A1, contributing to cyclin A1 accumulation, which led to promotion of cell proliferation in bladder cancer (39). Frizzled 8-associated APF (antiproliferative factor) maintained the stability of p53 via modulation of USP2a and murine double minute 2 (MDM2) (40). APF decreased USP2a expression and caused MDM2 ubiquitination, leading to inhibition of the interaction between p53 and MDM2, thereby impairing p53 ubiquitination (40). Overexpression of USP2a increased cell growth through upregulation of cyclin D1 at the mRNA and protein levels, while depletion of USP2a reduced cell proliferation in part via increased cellular p53 levels in T24 cells (40).

### USP21

USP21 has been gradually uncovered the essential role in carcinogenesis (41, 42). One integrative assay of 1q23.3 copy number gain in urothelial cancer patients with metastasis after platinum-based chemotherapy demonstrated that USP21, F11R, PPOX, DEDD, PFDN2 genes were closed linked to poor outcomes (43). Similarly, USP14 and USP21 were found to be associated with chemoresistance in bladder urothelial carcinoma with metastasis (44). Another study showed that USP21 expression was elevated in bladder cancer. High expression of USP21 was closely correlated with tumor metastasis and tumor size. Intriguingly, poorer survival rate was found in bladder cancer patients with higher levels of USP21 (45). In bladder cancer cells, increased expression of USP21 promoted cell proliferation, stimulated cell migration and invasion, enhanced tumor metastasis (45). Notably, overexpression of USP21 led to the development of EMT. Mechanistically, USP21 deubiquitinated EZH2 and stabilized its protein levels. USP21 could be a potential target for bladder cancer therapy (45). PD-L1 is observed in membrane of immune cells ad tumor cells. PD-L1 can bind to PD-1, leading to protection of tumor cells from an immune attack. The inhibitors of PD-1/PD-L1 can impair this binding and enhance the immune response against tumor cells (46, 47). USP21 has been identified to act as a deubiquitinase of PD-L1. Increased USP21 elevated PD-L1 abundance, whereas depletion of USP21 promoted PD-L1 degradation. Hence, targeting USP21 could be helpful to improve tumor immunotherapy (48).

### USP22

USP22 has been known to involve in tumor cell proliferation, invasion, stemness, cell cycle arrest, metastasis, immune response and drug resistance in human cancer (49). In bladder cancer, silencing USP22 by siRNAs induced cell cycle arrest and attenuated cell proliferation (50). USP22 siRNA transfection increased the expression of p53 and p21, decreased cyclin E expression in bladder cancer cells. Silencing of USP22 promoted the degradation of MDM2 in bladder cancer cells. USP22 siRNA transfection induced cell cycle at G0/G1 phase via upregulation of p53, p21 and downregulation of cyclin E in bladder cancer cells (50). Depletion of USP22 expression retarded the tumor growth of implanted bladder cancer cells in mice (50). Another study also revealed that USP22 depletion reduced cell cycle progression and retarded tumor growth in animal models of bladder cancer, liver cancer, lung cancer, breast cancer and ovarian cancer (51). USP22 has been reported to regulate immune evasion and drug sensitivity in cancer (52). USP22 has been identified to work as a new regulator of PD-L1. USP22 interacted with PD-L1 and maintained PD-L1 stability via deubiquitination in A549, H1299 and H1792 NSCLC cells (53). USP22 also interacted with CSN5 and kept its stability via deubiquitination. Either CSN5 or USP22 enhanced the binding of PD-L1 with the other one. The K6, K11, K27, K29, K33 and K63-linked ubiquitin chains were removed by USP22 in PD-L1 and CSN5 in HEK293FT cells. Hence, USP22 governed the PD-L1 protein levels via CSN5/PD-L1 pathway in HEK293FT cells (53). Silencing of USP22 enhanced T cell cytotoxicity and blocked lung tumorigenesis. This study showed a critical role of USP22 in regulation of immune evasion via maintenance of PD-L1 protein levels (53). It is required to define the role of USP22 in bladder tumorigenesis.

## E3 ubiquitin ligases in bladder cancer

### FBXW7

FBXW7 belongs to F-box protein family and shows a tumor suppressive function in cancer development (54). F-box proteins target numerous substrates and regulate proliferation, metastasis, EMT, cancer stem cells, and drug resistance (55–57). FBXW7 exhibited single nucleotide variants or insertion or deletion in non-schistosomiasis related-squamous cell carcinoma of urinary bladder (NSR-SCCUB) patients (58). NSR-SCCUB is not common type in urothelial carcinoma, which could have genomic alterations (58). FBXW7 targeted an epigenetic regulator ZMYND8 for ubiquitination and degradation in bladder cancer (59). ZMYND8 increased cell viability and colony formation, migrative ability in bladder cancer. FBXW7 interacted with and degraded ZMYND8 in a polyubiquitin-dependent manner. By a gene set enrichment analysis, ZMYND8 was observed to be positively correlated to tumor stemness markers, including FOXM1, SOX2 and NANOG (59). One group revealed that overexpression of p65 increased cell migration via FBXW7-induced ubiquitination and degradation of RhoGDIα protein in bladder cancer (60). RhoGDIα protein was found to be a p65 downstream target and mediated p65-induced cell migration in bladder cancer. Mechanistically, p65 enhanced FBXW7 stability via attenuating the mRNA transcription of PTEN (60). Hence, p65 inhibited PTEN mRNA transcription and subsequently promoted FBXW7 stability, leading to degradation of RhoGDIα in bladder cancer cells (60). Liu et al. found that upregulation of FBXW7 reduced the invasion and growth of bladder cancer cells, caused cell cycle arrest at G0/G1 phase. Increased FBXW7 activated GSK-3β phosphorylation and inhibited the expression of SREBP1a in bladder cancer cells (61). SREBP1 is a transcription factor, including two isoforms, SREBP-1a and SREBP-1c, which regulates the expression of lipogenesis genes. Studies have shown that SREBP1 regulates the expression of stearoyl-CoA desaturase, fatty acid synthase, and acetyl-CoA carboxylase (62). FBXW7 can bind with SREBP1a by a co-immunoprecipitation assay. *In vivo* study further validated the role of FBXW7 in regulation of SREBP1a (61). The role of FBXW7 in bladder cancer indicated that targeting FBXW7 is a novel approach for bladder cancer therapy.

### MDM2

MDM2 (mouse double minute 2 homologue) is involved in tumorigenesis mainly targeting p53 protein in different cancer types, including bladder cancer (63, 64). In 1994, upregulation of MDM2 and p53 expression was observed in bladder cancer patients (65). Moreover, p53 and MDM2 were found to be key factors in the progression of bladder cancer (66). There was an association between TP53 (codon 72, arginine> proline), MDM2 (SNP309, T>G) polymorphisms and patient’s survival in bladder cancer after chemoradiotherapy (CRT) (67). Patients with MDM2 T/G + G/G genotypes exhibited a good survival rate after CRT. TP53 and MDM2 with more than two of variant alleles exhibited an improved survival (67). For example, MDM2 SNP309 G-variant was revealed to be correlated with tumor cell invasive growth and the risk of bladder cancer (68, 69). Mao et al. found that OCT3/4 increased tumor immune escape via upregulation of TET1 and NRF2 expression, leading to enhancement of MDM2 expression, which contributed to acceleration of tumor immune evasion in bladder cancer (70). Small-molecule MDM2 inhibitors have been detected in clinical trials for improving the efficacy of cancer treatment (71, 72). MDM2 inhibitor APG-115 was reported to enhance the efficacy of PD-1 blockade via increasing anticancer immunity in the tumor microenvironment (73). One MDM2 inhibitor, AMG-232, sensitized tumor cells to T-cell-induced killing in tumors with high expression of MDM2 (74). The MDM2 ligand Nutlin-3 modulated the expression of PD-L1 and CD276 (75). Nutlin-3 induced the expression of PD-L1, while MDM2 did not bind PD-L1 (75). Suppression of MDM2 by HDM201 inhibitor facilitated anticancer responses via interaction with the stromal and immune microenvironment in tumor cells with p53 wild-type (76). MDM2 gene amplification could be a useful biomarker for prediction of a better response for targeted therapies in PD-L1 positive or negative urothelial bladder cancer (77).

### TRIM38

TRIM38 functions as a SUMO ligase or an E3 ubiquitin ligase and targets several cellular signaling components (78). Glucose transporter type 1 (GLUT1) was upregulated in bladder cancer and correlated with poor survival rate and poor prognosis in patients with bladder cancer (79, 80). Moreover, GLUT1 was identified as an independent biomarker for prognosis in bladder cancer patients after radical cystectomy treatment (81). GLUT1 was also taken part in cisplatin resistance in bladder cancer, which can be regulated by miR-218 (82). According to TCGA bladder cancer database, TRIM38 expression was low in bladder cancer patients. Lower expression of TRIM38 was linked to shorter survival rate and worse prognosis in patients with bladder cancer (83). TRIM38 was further found to regulate proliferation, stemness and invasion of bladder cancer cells. Strikingly, TRIM38 had an interaction with GLUT1 and enhanced the ubiquitination and degradation of GLUT1 in bladder cancer cells. Accordingly, BAY-876, an inhibitor of GLUT1, inhibited proliferation and tumor growth in bladder cancer cells and mouse models (83).

## Other deubiquitinases and E3 ubiquitin ligases

Accumulating evidence has shown that many E3 ubiquitin ligases are involved in bladder tumorigenesis. For instance, the E3 ubiquitin ligase cIAP2 (cellular inhibitor of apoptosis protein 2) was elevated after inhibition of histone deacetylase (HDAC) in bladder cancer. MRE11, which regulates DNA repair pathways and double-strand breaks, was also inhibited by HDAC inhibitors (84). The cIAP2 was found to bind with MRE11 and governed radio-sensitization after HDAC inhibitor treatment. cIAP2 modulated the ubiquitination of MRE11 and caused the downregulation of MRE11 in bladder cancer cells (84). Therefore, cIAP2 might be a promising target for improving chemoradiation strategy in bladder cancer. Suppression of GRIM19 expression impaired ubiquitination-mediated degradation of Bcl-xL in bladder cancer cells, conferring to promotion of cisplatin chemoresistance (85). Overexpression of GRIM19 potentiated cisplatin sensitivity and reduced the invasion and proliferation of bladder cancer cells, which was due to attenuation of Bcl-xL polyubiquitination and degradation (85).

Yes-associated protein (YAP) is one of key effectors in the Hippo tumor suppressor pathway, which regulates organ size and tissue growth and tumorigenesis (86, 87). Luo et al. reported that MINDY1, a DUB enzyme, interacted with YAP and acted as a deubiquitylase of YAP to stabilize YAP protein levels in bladder cancer (88). Consistently, silencing of MINDY1 reduced proliferation of bladder cancer cells. Overexpression of YAP abrogated the MINDY1 depletion-induced inhibition of cell proliferation in bladder cancer cells (88). Connective tissue growth factor (CTGF) controls differentiation, adhesion and proliferation, and involves in Hippo pathway, NF-κB and p53 pathways, leading to regulation of cancer, inflammation and fibrosis (89). Cysteine-rich protein 61 (CYR61) was reported to involve in the development of melanoma (90), glioma (91) and esophageal squamous cell carcinoma (92). Exosomal miR-217 mimic promoted migration and proliferation in 5637 and T24 cells via upregulation of YAP and its targets, such as CTGF, CYR61 and ANKRD1 (93). Downregulation of MINDY1 disrupted the YAP stabilization and inhibited the expression of YAP downstream genes, such as CTGF, CYR61 and ANKRD1 in bladder cancer (88). MINDY1 could be a possible biomarker and therapeutic target for bladder cancer (88). RNF126 (ring finger protein 126), acting as a E3 ubiquitin ligase, has been reported to be overexpressed in numerous cancer types and correlated with tumorigenesis (94). RNF126 expression was elevated in bladder cancer tissues via a TCGA database analysis. Depletion of RNF126 remarkably impaired proliferation and metastasis of bladder cancer cells via modulation of the EGFR/PI3K/AKT pathway. RNF126 silencing reduced EGFR expression and AKT phosphorylation, slightly inhibited PI3K expression, and remarkably increased the PTEN protein levels in UMUC3 and T24 cells. The mRNA levels of AKT and EGFR were reduced after RNF126 downregulation, but PTEN mRNA levels did not change in RNF126-silencing cells. Notably, PTEN was identified as a new substrate of RNF126 (95). RNF126 bound to PTEN and led to polyubiquitination and degradation of PTEN. Inhibition of RNF126 oncoprotein could be a novel approach for bladder cancer therapy (95). It has been known that c-Cbl is an E3 ubiquitin ligase that targets its substrates for degradation (96). C-Cbl was reported to target the EGFR for ubiquitination and degradation (97). Another study revealed that USP8 can regulate SOX2 ubiquitination and degradation in bladder cancer (98).

## Deubiquitinases and E3 ubiquitin ligases regulate immunotherapy

The E3 ubiquitin ligases have been approved as important factors to govern the tumor microenvironment and affect immunotherapy in human cancers (99). Evidence has dissected that the E3 ubiquitin ligases control PD-1/PD-L1 protein levels and enhance tumor immunotherapy (100). For example, FBXO38, FBXW7 and C-Cbl target PD-1, whereas SPOP and FBXO22 target PD-L1. In addition, USP7, USP8 and USP22 target PD-L1 to maintain the PD-L1 protein levels (100, 101).

## RNF144A regulates PD-L1

RNF144A is an E3 ubiquitin ligase for the degradation of DNA-PKcs (DNA-dependent protein kinase catalytic subunit), leading to promotion of apoptosis during DNA damage (102, 103). RNF144A governed PARP inhibitor sensitivity via targeting PARP1 in ubiquitin-dependent manner in breast cancer cells (104). In addition, RNF144A expression was decreased due to promoter hypermethylation in breast cancer cells (105). Moreover, RNF144A targeted the stability of HSPA2 via ubiquitin-dependent regulation in breast cancer (106). Furthermore, RNF144A degraded YY1 and inhibited the expression of GMFG as well as suppressed oncogenesis in breast cancer (107). RNF144A maintained the activation of EGFR signaling pathway to enhance EGF-involved cell proliferation (108). RNF144A controlled the stability of LIN28B *via* the uniquitin-proteasome manner and inhibited stem cell properties in ovarian cancer cells (109).

In bladder cancer cells, depletion of RNF144A elevated the stabilization of PD-L1 protein and enhanced carcinogen-mediated bladder oncogenesis (110). Mice with RNF144A deficiency were more prone to initiation of bladder cancer after carcinogen exposure. RNF144A knockout mice displayed the higher expression of PD-L1. RNF144A can bind with PD-L1 and enhanced ubiquitination and disruption of PD-L1 in the intracellular vesicles and plasma membrane (110). RNF144A depletion in mice caused a decrease of tumor infiltration CD8+ T-cells in the carcinogen-induced bladder cancer. Moreover, RNF144A depletion stimulated cellular differentiation, showing that a luminal subtype marker GATA3 was increased in RNF144A knockout tumors (110). This phenotype could be due to that RNF144A maintained EGFR expression. Hence, depletion of RNF144A increased the expression of PD-L1, DNA-PKcs and BMI1, resulting in the carcinogen-mediated the development of bladder cancer (110).

## NEDD4 regulates PD-L1

An E3 ubiquitin ligase NEDD4 (also known as NEDD4-1) belongs to NEDD4 family, which has shown a critical function in carcinogenesis and progression (111, 112). NEDD4 performs its biological functions via targeting numerous substrates for ubiquitination and degradation (113, 114). NEDD4 has been revealed to regulate many functions, including growth, cell cycle, proliferation, differentiation, invasion, motility, apoptosis, necrosis, autophagy and metastasis (115). NEDD4 has been identified to take part in bladder cancer initiation and development. Inhibition of LAPTM5 blocked cell viability and growth and caused cell cycle arrest at G0/G1 phase via inhibition of p38 and ERK1/2 activation in bladder cancer (116). Depletion of NEDD4 suppressed the transportation of LAPTM5 from Golgi to lysosome, which could affect bladder tumorigenesis (116). Suppression of NEDD4 displayed antitumor activity in bladder cancer cells (117). Mao et al. found that NEDD4 can bind to KLF8 (Kruppel-like factor 8) and target the miR-132 and NRF2 (nuclear factor E2-related factor 2) axis in bladder cancer, contributing to acceleration of tumor growth, recurrence and lung metastasis (118). NEDD4 depletion reduced K63-linked polyubiquitination of KLF8 and inhibited the stability and transcriptional ability of KLF8 (118). NEDD4 promoted the interaction between KLF-8 and miR-132 promoter region, resulting in suppression of miR-132. Moreover, miR-132 inhibited the expression of NRF2 in bladder cancer cells, leading to repression of cell migration and viability (118).

Fibroblast growth factor receptor 3 (FGFR3) has been known to play a key role in bladder cancer development. FGFR3 rearrangements and missense mutations were reported in bladder cancer (119). One study showed that suppression of FGFR3 increased PD-L1 protein levels in FGFR3-expressing bladder cancer due to influencing its ubiquitination, leading to suppression of the anticancer activity of CD8+ T cells. FGFR3 expression was negatively associated with PD-L1 expression levels in bladder cancer tissues. FGFR3 activation can promote NEDD4 phosphorylation. NEDD4 catalyzed K48-linked polyubiquitination of PD-L1 *via* their interactions. CD8+ T-cell infiltration and anticancer ability were largely impaired because of upregulation of PD-L1 in bladder tumor cells in mice with NEDD4 knockout bladder cancer. Targeting FGFR3 and PD-L1 increased CD8+ T-cell-induced anticancer efficacy and exhibited effective tumor suppression in bladder cancer. This work provided a molecular clue among NEDD4, PD-L1 and FGFR3, suggesting that targeted therapy in combination with immune therapy could be much better for the treatment of bladder cancer. Therefore, NEDD4 targets PD-L1 for ubiquitination and destruction in FGFR3-overexpressing bladder cancer, indicating that NEDD4 is associated with immune surveillance *via* regulation of PD-L1 in bladder cancer (120). One group showed that a natural compound lycorine downregulated the expression of NEDD4 in bladder cancer, leading to suppression of cell growth and invasiveness (121). Hence, natural compounds targeting NEDD4 could be useful to improve immunotherapy in bladder cancer.

## USP7 regulates PD-L1 expression

USP7 (ubiquitin-specific protease 7), also named as HAUSP (herpesvirus-associated protease), has been discovered to be associated with oncogenesis in some cancer types, including bladder cancer (122–125). USP7 has been revealed to control the anti-tumor immune responses. Inhibition of USP7 by its inhibitors impedes the activity of Treg cells, enhances polarization of tumor-related macrophages in tumor cells (126). It has been reported that USP7 modulated the expression levels of CCDC6 in bladder cancer. One USP7 inhibitor, P5091, regulated CCDC6 degradation and enhanced cell sensitivity to PARP inhibitors. Combined therapy with DNA damage inducer RRx-001 and P5091 promoted the tumor cell sensitivity to PARP inhibitors (127).

DNA methylation is regulated by DNMTs (DNA methyltransferases). SB216763, an inhibitor of GSK3 (glycogen synthase kinase-3), increased cell proliferation and upregulated the expression of pGSK3β, β-catenin and DNMT1 (128). The expression of USP7, DNMT1, UHRF1 and β-catenin was inhibited after re-expression of WIF-1 and treatment with DNMT1 inhibitor DAC (128). One study revealed that PD-L1 expression was positively associated with USP7 levels in gastric cancer patients. USP7 directly bound to PD-L1 and stabilize it (129). Abrogation of USP7 impaired the interaction between PD-1 and PD-L1, leading to sensitization of cancer cells to T cell killing in cancer cells and in mice. In addition, inhibition of USP7 by its inhibitor reduced cell proliferation due to p53 stabilization in gastric cancer cells (129). Hence, USP7 suppression by its inhibitors not only blocked gastric tumor cell proliferation but also inhibit the expression of PD-L1 to improve anti-cancer immune response in gastric cancer (129). It is required to explore whether USP7 inhibitors could enhance the immune response of bladder cancer. USP7 inhibitors have been developed to perform anticancer ability in various cancer types (130). It is necessary to determine whether these USP7 inhibitors can improve immunotherapy in bladder cancer.

## Other E3 ubiquitin ligases regulate immunotherapy

One group used TCGA and GEO database to analyze ubiquitination-related molecular subtypes for bladder cancer (131). This group found a total of four ubiquitination-related molecular subtypes of bladder cancer. These four subgroups had various tumor microenvironment, prognosis, clinical characteristics and PD-L1 expression level. In addition, six ubiquitination-related genes (URGs), including HLA-A, UBE2D1, UBE2T, USP5, TMEM128 and UBE2N, could be useful for prognostic markers (131).

## Compounds regulate E3 ligases in bladder cancer

In recent years, some compounds have been uncovered to regulate the expression of E3 ubiquitin ligases in human malignancies, including bladder cancer (132–134). β-lactam cephalosporin antibiotic cefepime has been uncovered to deplete PD-L1 and promote tumor DNA damage and increase sensitivity of DNA-damaging compounds in multiple tumor cell lines, such as bladder cancer, melanoma, GBM (glioblastoma multiforme) and ovarian cancer (135). Cefepime inhibited tumor PD-L1 via regulation of its ubiquitination, enhanced efficacy of DNA-damaging compounds in mice, stimulated immunogenic tumor STING pathway. Ceftazidime exhibited the similar performance as cefepime in regulation of PD-L1 and DNA-damaging agent therapeutic efficacy. Taken together, cefepime and ceftazidime could improve immunotherapy and DNA-damaging agent efficacy in bladder cancer (135). Hispolon from Phellinus linteus is a natural polyphenol and conducted a function as a cancer killer via targeting several signaling pathways (136). Hisplon inhibited tumor cell growth via upregulation of p21 in bladder cancer cells (137). Hispolon promoted the ubiquitination and degradation of MDM2 in bladder cancer cells. ERK1/2 was activated and recruited to MDM2 and led to MDM2 ubiquitination. Inhibition of ERK1/2 by U0126 blocked hispolon-mediated caspase-7 cleavage. Hence, hispolon downregulated MDM2 via degradation in bladder cancer (137).

Allyl isothiocyanate was often obtained from cruciferous vegetables and caused mitotic arrest via upregulation of ubiquitination and degradation of alpha and beta-tubulin in bladder cancer cells (138). PR-619 was an inhibitor of deubiquitylating enzymes and overcame cisplatin resistance via the inhibition of c-Myc in bladder urothelial carcinoma cells (44). Stevioside was identified by high-throughput screening as a useful compound to increase cell apoptosis via activation of GSK-3β and induction of FBXW7, contributing to downregulation of MCL-1 in bladder cancer (139). Similarly, OSU-T315, an inhibitor of integrin-linked kinase, was observed to inhibit Mcl-1 expression levels via targeting the GSK-3β/FBXW7 axis in bladder cancer cells (140). Green tea polyphenol EGCG plays a tumor suppressive role in bladder cancer via inactivation of NF-kappa B. Moreover, EGCG promoted the anticancer activity of doxorubicine via modulation of NF-κB/MDM2/p53 pathway in bladder cancer (141). Proguanil, which is often used as an anti-malarial drug, inhibited the cell growth by promotion of EGFR degradation and induction of autophagy in bladder cancer (97). Proguanil enhanced the interaction between EGFR and Caveolin-1, leading to endocytosis and recruiting c-Cbl to elevate EGFR degradation via the lysosomal pathway (97). 4-hydroxynonenal (HNE), a pro-oxidant compound, conducted tumor suppressive function via altering several signaling pathways. HNE upregulated YAP phosphorylation and ubiquitination, caused promotion of YAP proteasomal degradation in bladder cancer cells (142). One compound ChlA-F blocked cell invasion via inhibition of SOX2 protein by USP8-mediated SOX2 degradation in bladder cancer (98). Therefore, compounds can regulate E3 ubiquitin ligases to enhance the ubiquitination and degradation of specific targets, which lead to antitumor activity in bladder cancer (**Table 1**).

**Table 1:** Compounds regulate E3 ligases in bladder cancer.

Item	Target	Function	Ref.
Cefepime	PD-L1 ubiquitination, activation of STING.	Enhances efficacy of DNA-damaging compounds	(135)
Ceftazidime	PD-L1 ubiquitination	Increases immunotherapy and DNA-damaging agent efficacy	(135)
Hispolon	MDM2 ubiquitination and degradation, p21 upregulation.	Inhibits tumor cell growth	(137)
Allyl isothiocyanate	Alpha and beta-tubulin ubiquitination and degradation	Causes mitotic arrest	(138)
PR-619	Inhibits c-Myc expression	Overcomes cisplatin resistance	(44)
Steviosode	Activates GSK-3β/FBXW7, inhibits Mcl-1.	Increases cell apoptosis	(139)
OSU-T315	Inhibits Mcl-1, targets GSK-3β/FBXW7	Reduces cell growth and increases apoptosis	(140)
EGCG	Targets NF-κB/MDM2/p53	Increase antitumor activity of doxorubicine	(141)
Proguanil	Promotes EGFR degradation	Induces autophagy	(97)
HNE	Upregulates YAP phosphorylation and ubiquitination and degradation	Performs tumor suppressive function	(142)
ChlA-F	Inhibits SOX2 *via* USP8-mediated degradation	Blocks cell invasion	(98)

## Noncoding RNAs target E3 ligases

Multiple studies have shown that noncoding RNAs govern carcinogenesis in bladder cancer (143–146). Noncoding RNAs are transcribed from DNA, but not translated into proteins, including microRNAs (miRNAs), lncRNAs (log noncoding RNAs), siRNAs (small interfering RNAs), snRNAs (small nuclear RNAs) and piRNAs (147–149). Noncoding RNAs target E3 ubiquitin ligases to control bladder cancer initiation and progression. For example, miR-143 inhibited the expression of MDM2 and performed a tumor suppressive function via inhibition of cell growth and migration in bladder cancer (150). LncRNA SNHG1 sponged miR-9-3p expression and upregulated the expression of MDM2 in bladder cancer cells. MDM2 targeted PPARγ for ubiquitination and degradation, leading to facilitating the development of bladder cancer (151). LncRNA LNPPS displayed a tumor suppressive function via modulation of MDM2/p53 degradation in bladder cancer (152). LncRNA SNHG18 was downregulated in tumor specimens of bladder cancer patients. The bladder cancer patients with high expression of SNHG18 had a better survival rate. Upregulation of SNHG18 reduced proliferation of bladder cancer cells and decreased tumor sizes in mice (153). SNHG18 impaired the expression of c-Myc via targeting its ubiquitination and degradation, resulting in p21 upregulation in bladder cancer (153).

**Figure 1:**
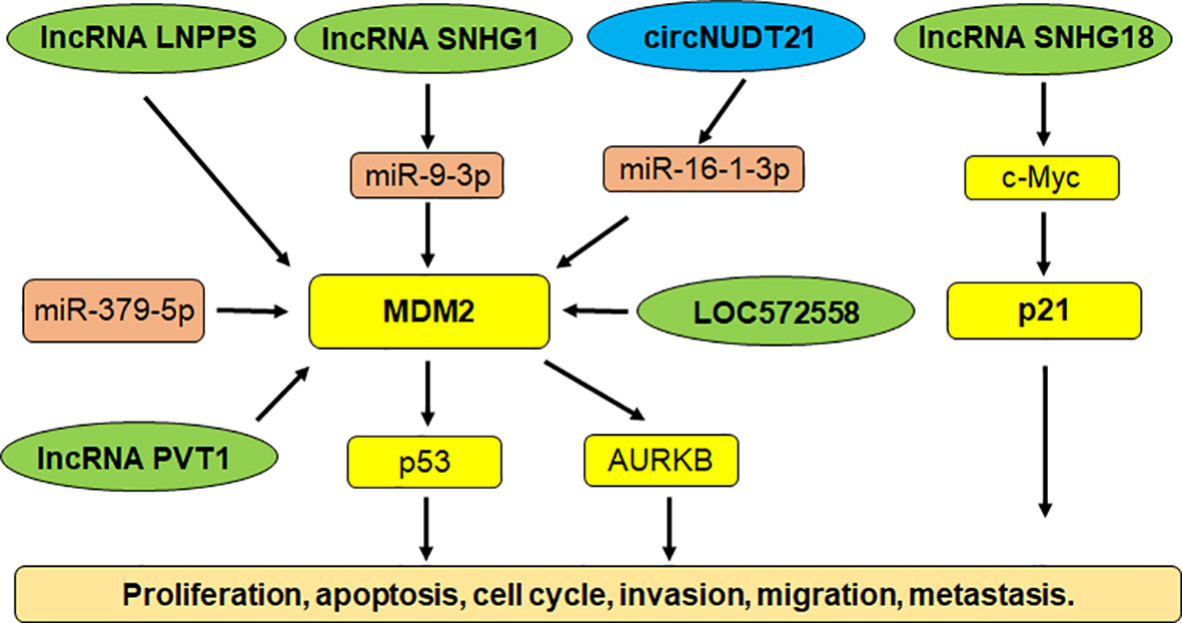
Noncoding RNAs regulate E3 ubiquitin ligases in bladder cancer.

LncRNA PVT1 promoted the expression of MDM2 and accelerated the p53 ubiquitination and degradation, leading to promoting cell invasion and cell resistance to doxorubicin (154). AURKB (Aurora kinase B) was increased after MDM2 upregulation induced by lncRNA PVT1 in bladder cancer cells. AURKB further promoted the p53 ubiquitination that was induced by MDM2 (154). LncRNA LOC572558 overexpression was downregulated in tumor tissues of bladder cancer patients. In T24 and 5637 bladder tumor cells, upregulation of LOC572558 suppressed cell growth and invasion, induced apoptosis and caused cell cycle arrest, which was correlated with p53 phosphorylation, MDM2, AKT dephosphorylation (155). Chen et al. reported that a circRNA circNUDT21 altered the miR-16-1-3p/MDM2/p53 axis and accelerated tumor progression in bladder cancer (156). Hence, noncoding RNAs are pivotal to regulate E3 ubiquitin ligases in bladder tumorigenesis (**Figure 1**).

## Conclusions and future perspectives

**Figure 2:**
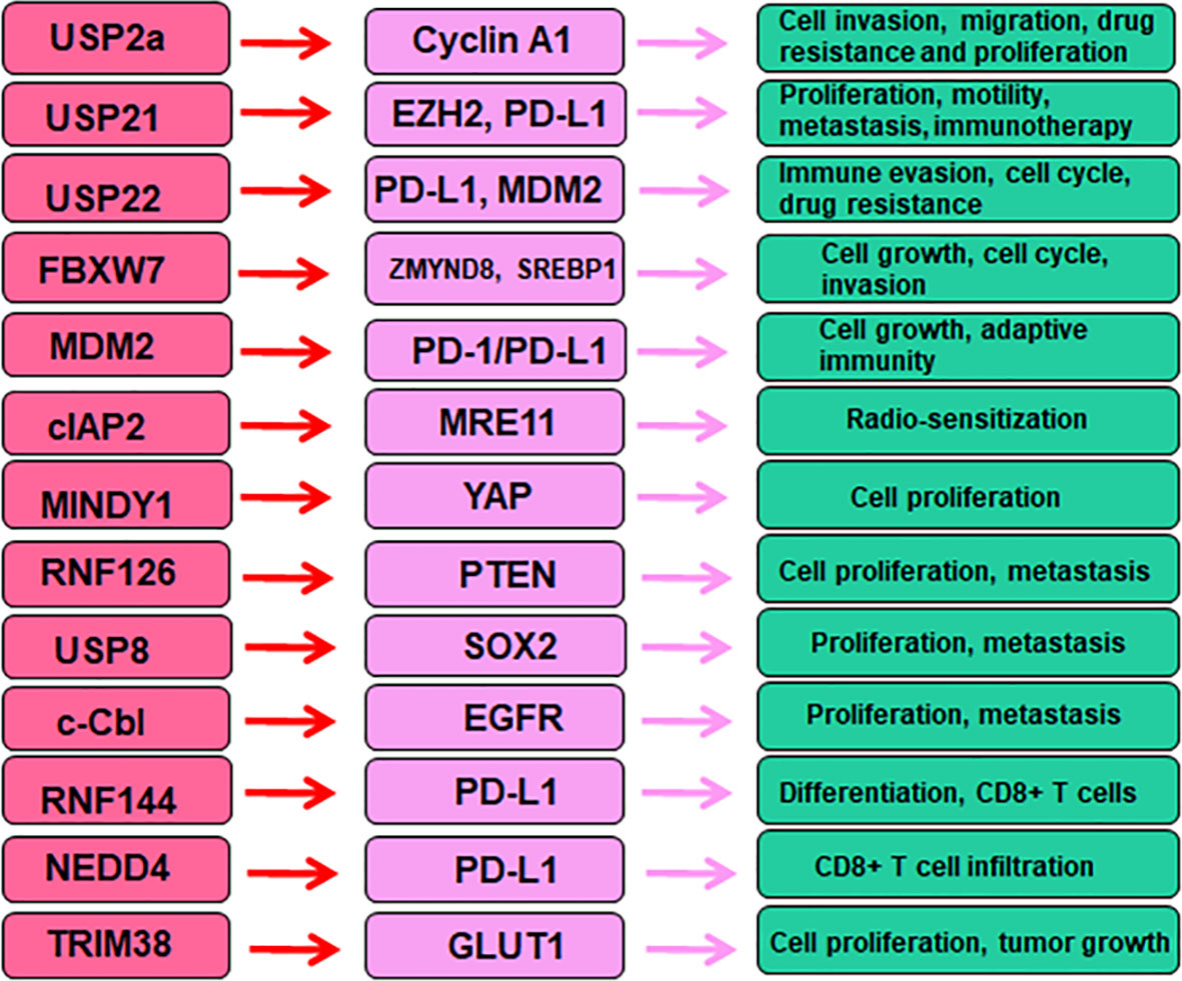
E3 ubiquitin ligases regulate several proteins in bladder cancer.

In conclusion, E3 ubiquitin ligases are critical in bladder cancer initiation and development via targeting specific substrates. E3 ubiquitin ligases alter tumor immunotherapy and drug resistance in bladder cancer (**Figure 2**). Targeting E3 ubiquitin ligases could be an effective strategy for bladder cancer therapy. It is necessary to mention several points regarding the roles of E3 ubiquitin ligases in bladder cancer. First, besides ubiquitination, there are many other types of PTMs to involve in bladder tumorigenesis. For example, activation of autophagy altered acetylation profile relevant for mechanotransduction in bladder tumor cells (157). PD-L1 methylation was found to be an independent biomarker for patient survival in bladder cancer (158). Histone demethylase JMJD1A promoted glycolysis via coactivation of HIF1α and led to promotion of urinary bladder cancer progression (159). SIRT1 (silent information regulator sirtulin 1), a NAD+ dependent deacetylase, elevated the expression of GLUT1 and stimulated tumor progression in bladder cancer via modulation of glucose uptake (160).

Second, in addition to E3 ubiquitin ligases, E2 enzyme has also been involved in bladder carcinogenesis. Ubiquitin-conjugating enzyme E2S (UBE2S) is a type of E2 enzyme in the ubiquitin system, which has displayed several activities in carcinogenesis (161). UBE2S has been suggested to promote the ovarian cancer development via targeting PI3K/AKT/mTOR pathway and modulating cell apoptosis and cell cycle (162). UBE2S reduced cell chemosensitivity via regulation of PTEN-AKT pathway in hepatocellular carcinoma (163). UBE2S expression was increased in urinary bladder cancer cells. Knockdown of UBE2S led to reduction of proliferation and induction of cell apoptosis, while upregulation of UBE2S resulted in an inverse phenotype in bladder cancer cells (164). Moreover, UBE2S performed the oncogenic functions via modulation of the mTORC1 pathway in bladder cancer cells. UBE2S targeted tuberous sclerosis 1 (TSC1) for ubiquitous degradation (164). Collectively, UBE2S promoted bladder cancer progression via degradation of TSC1 and activation of mTOR signaling pathway.

Third, noncoding RNAs have been identified as potential biomarkers for bladder cancer prognosis (165, 166). Besides lncRNAs, miRNAs and circRNAs, one study showed that PIWI-interacting RNAs (piRNAs) and snRNAs are important in bladder carcinogenesis (167, 168). In this work, it has been shown that 106 piRNAs were increased and 91 piRNAs were decreased in bladder tumor specimens. Upregulation of piRABC reduced proliferation, colony formation, but enhanced cell apoptosis in bladder cancer cells. Moreover, piRABC increased the expression of TNFSF4 protein in bladder cancer cells (167). Fourth, several F-box proteins have been described to target PD-1/PD-L1 in cancers; however, whether other F-box proteins can regulate immunotherapy is unclear. For example, FBXO45 has shown an essential role in tumorigenesis and malignant progression (169–171). FBXO22 targeted PD-L1 for degradation and sensitized tumor cells to DNA damage (172). FBXO1, FBXO20, FBXO22, FBXO28, FBXO32 and FBXO45 have been found to be associated with immune infiltration in pancreatic cancer (173). Hence, it is required to explore whether these F-box proteins are involved in immunotherapy in bladder cancer.

Fifth, it has been validated that PROTACs are novel tools for the enhancement of immunotherapy in human cancers (174). PROTACs have been designed to degrade a protein of interest (POI), resulting in a reduction of the expression of the POI (175, 176). One study has shown that one BET (bromodomain and extraterminal domain) inhibitor mivebresib synergized with a Bcl-xL PROTAC degrader PZ703b increased cell apoptosis through the mitochondrial pathway in bladder cancer (177). Another study showed that BRD4 PROTAC degrader QCA570 increased the degradation of BRD4 protein, leading to induction of cell apoptosis and cell cycle arrest, which caused antiproliferation ability in bladder cancer (178). All in a word, E3 ubiquitin ligases are essential for the initiation and progression of bladder cancer. Regulation of E3 ubiquitin ligases might be a potential therapeutic strategy for bladder cancer treatment.

## Data availability statement

The data in this study are available from the corresponding author on reasonable request.

## Author contributions

XMW and YZ wrote the manuscript. YW and HC made the tables and figures. XJW edited and revised the manuscript. All authors contributed to the article and approved the submitted version.
